# Genome-Wide Analysis of MicroRNAs and Their Target Genes Related to Leaf Senescence of Rice

**DOI:** 10.1371/journal.pone.0114313

**Published:** 2014-12-05

**Authors:** Xiangbin Xu, Haiqi Bai, Chaoping Liu, Eryong Chen, Qifeng Chen, Jieyun Zhuang, Bo Shen

**Affiliations:** 1 College of Life and Environmental Sciences, Hangzhou Normal University, Hangzhou, China; 2 State Key Laboratory of Rice Biology, China National Rice Research Institute, Hangzhou, China; National Taiwan University, Taiwan

## Abstract

Grain production of rice (*Oryza sativa* L.) is a top priority in ensuring food security for human beings. One of the approaches to increase yield is to delay leaf senescence and to extend the available time for photosynthesis. MicroRNAs (miRNAs) are key regulators of aging and cellular senescence in eukaryotes. Here, to help understand their biological role in rice leaf senescence, we report identification of miRNAs and their putative target genes by deep sequencing of six small RNA libraries, six RNA-seq libraries and two degradome libraries from the leaves of two super hybrid rice, Nei-2-You 6 (N2Y6, age-resistant rice) and Liang-You-Pei 9 (LYP9, age-sensitive rice). In total 372 known miRNAs, 162 miRNA candidates and 1145 targets were identified. Compared with the expression of miRNAs in the leaves of LYP9, the numbers of miRNAs up-regulated and down-regulated in the leaves of N2Y6 were 47 and 30 at early stage of grain-filling, 21 and 17 at the middle stage, and 11 and 37 at the late stage, respectively. Six miRNA families, osa-miR159, osa-miR160 osa-miR164, osa-miR167, osa-miR172 and osa-miR1848, targeting the genes encoding APETALA2 (AP2), zinc finger proteins, salicylic acid-induced protein 19 (SIP19), auxin response factors (ARF) and NAC transcription factors, respectively, were found to be involved in leaf senescence through phytohormone signaling pathways. These results provided valuable information for understanding the miRNA-mediated leaf senescence of rice, and offered an important foundation for rice breeding.

## Introduction

MicroRNAs (miRNAs) are short, single strand and endogenous non-coding small RNAs (sRNAs) that negatively regulate gene expressions at post-transcriptional level by repressing gene translation or degrading target mRNAs [1], [2]. They are encoded by independent transcriptional units in intergenic regions and transcribed by RNA polymerase II or III to form primary miRNA (pri-miRNA). The pri-miRNA are processed by ribonuclease III enzymes into a stem–loop miRNA::miRNA* duplex [3]. In plants, the stem loop region of pri-miRNAs is processed by Dicer-like (DCL) endonuclease, forming small double stranded RNA (dsRNA) miRNA: miRNA* [2], [3]. The mature miRNA is incorporated in the RNA-induced silencing complex (RISC) with endonuclease AGO and guide the cleavage or translational repression of the target mRNA by complementary base-pairing [4]. In plants, miRNAs are involved in multiple crucial biological processes [5], [6], such as organ development and plant responses to environmental stresses [7]–[9]. Notably, miRNAs also play important roles in plant senescence. In an ethylene-dependent manner, the down-regulated miR164 increased the transcripts of *NAC1*, *ORE1* (*AtNAC2*) and *At5g61430*, and positively regulated cell death and senescence during leaf aging of *Arabidopsis thaliana* [10]. On the other hand, miR164 ectopic expression or lack of ORE1 activity promoted longevity of *Arabidopsis* leaf [10]. Another miRNA, miR319, targeting *TB1*, *CYC* and *PCF* (*TCP*), has crucial function in repressing the onset of senescence. Overexpression of miR319 caused plants to stay green much longer while compromised miR319-dependent regulation of one of its main targets, TCP4, resulted in increased expression of genes that are normally active in older leaves [11]. Moreover, disrupting miR394 expression was reported to lead to leaf development abnormalities. Defects with upward curling leaves were observed in transgenic plants overexpressing MIR394a/b, whereas loss of MIR394 function resulted in a curled-down leaf phenotype. The mechanism mainly was the interplay between miR394 and its target LCR (*LEAF CURLING RESPONSIVENESS*), an F-box (SKP1-Cullin/CDC53-F-box) gene, which involved in regulating leaf curling-related morphology through auxin [12].

To date, 30,424 miRNAs from 206 species (miRbase 20, http://www.mirbase.org/) are identified, but the function for most miRNAs remains unknown. Identification of their targets is a primary component for understanding their biological functions in diverse biological processes. Recently development of degradome sequencing provided a new strategy for validating the splicing targets on a whole genome scale, which revolutionized the traditional computational target prediction and has been successfully employed on identifying miRNA targets in plants [13]–[15].

Rice (*Oryza sativa* L.) is one of the most important food crops, which provides the staple for over half of the world's population. Demand for rice continues to increase since the world consumption has exceeded production [16]. Hence, enhancing rice production for ensuring food security is one of the top priorities for human being. However, leaf aging or senescence might seriously affect the grain yield of a rice variety. For example, Liang-You-Pei 9 (LYP9), one of the super hybrid rice, possesses big advantages of high yield and high disease resistance [17], but is age-sensitive [18], which often decreases grain yield. Nei-2-You 6 (N2Y6), one of the other super hybrid rice, shows characteristics of age-resistant and often with high grain yield [19]. Understanding the complex mechanism of leaf senescence will help to mitigate yield reduction by prolonging leaf life. To have a better understanding of the complex mechanism of leaf senescence, miRNAs and their targets involved in leaf senescence of N2Y6 and LYP9 were analyzed by high-throughput sequencing in the present study.

## Results

### Senescence characterization of leaves in two hybrid rice

**Figure 1:**
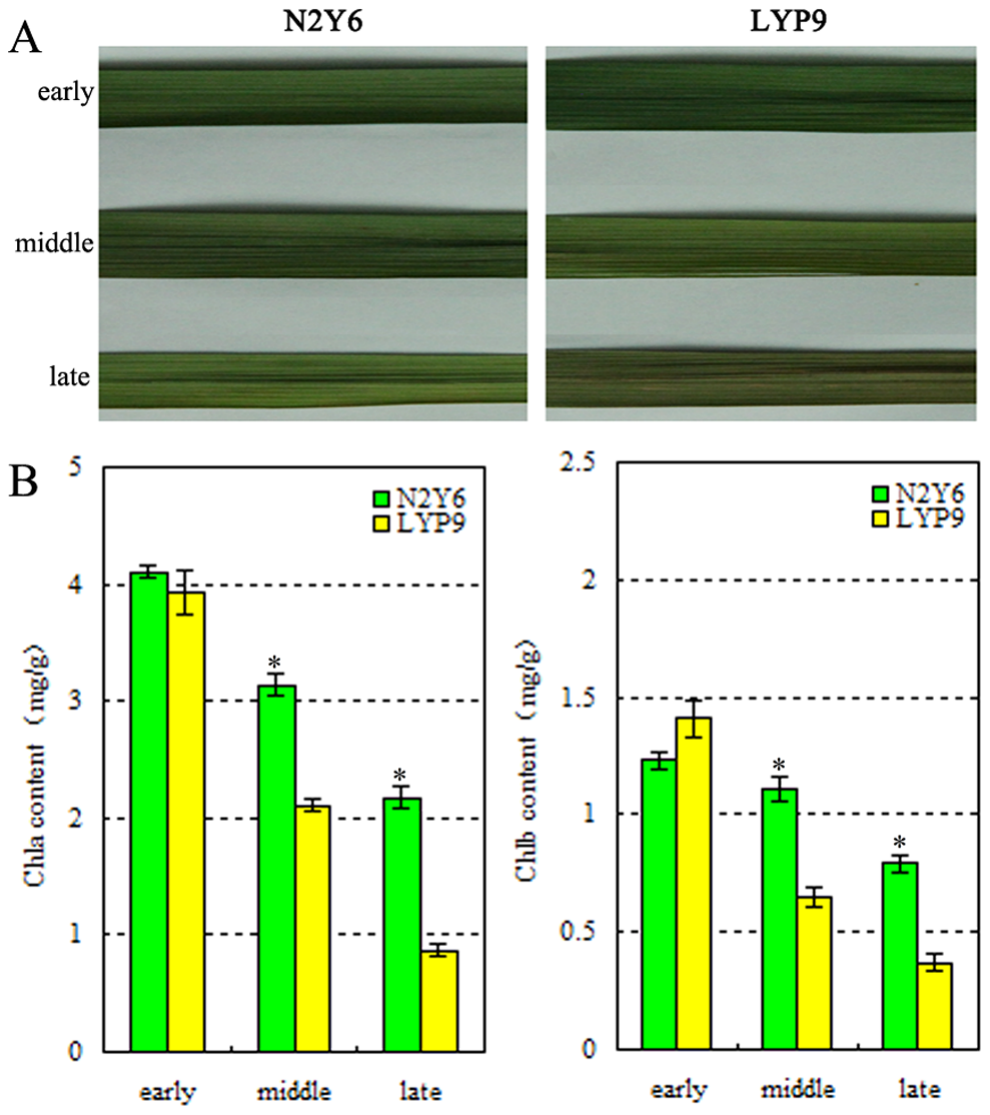
Senescence characterization of the leaves at the early, middle and late stages of grain-filling in LYP9 and N2Y6. (A) Senescence phenotype of the leaves; (B) The content of chlorophyll a and chlorophyll b in leaves.

From the senescence phenotype of leaves at the early, middle and late stages of grain-filling in LYP9 and N2Y6 (Fig. 1 A), N2Y6 were found to be more age-resistance than LYP9. The content of chlorophyll a and chlorophyll b in leaves at the early, middle and late stages of grain-filling of N2Y6 was higher than that of LYP9 (Fig. 1 B). These characterizations further confirmed that N2Y6 was age-resistance and LYP9 was age sensitive.

### Overview of deep sequencing in small RNA

**Figure 2:**
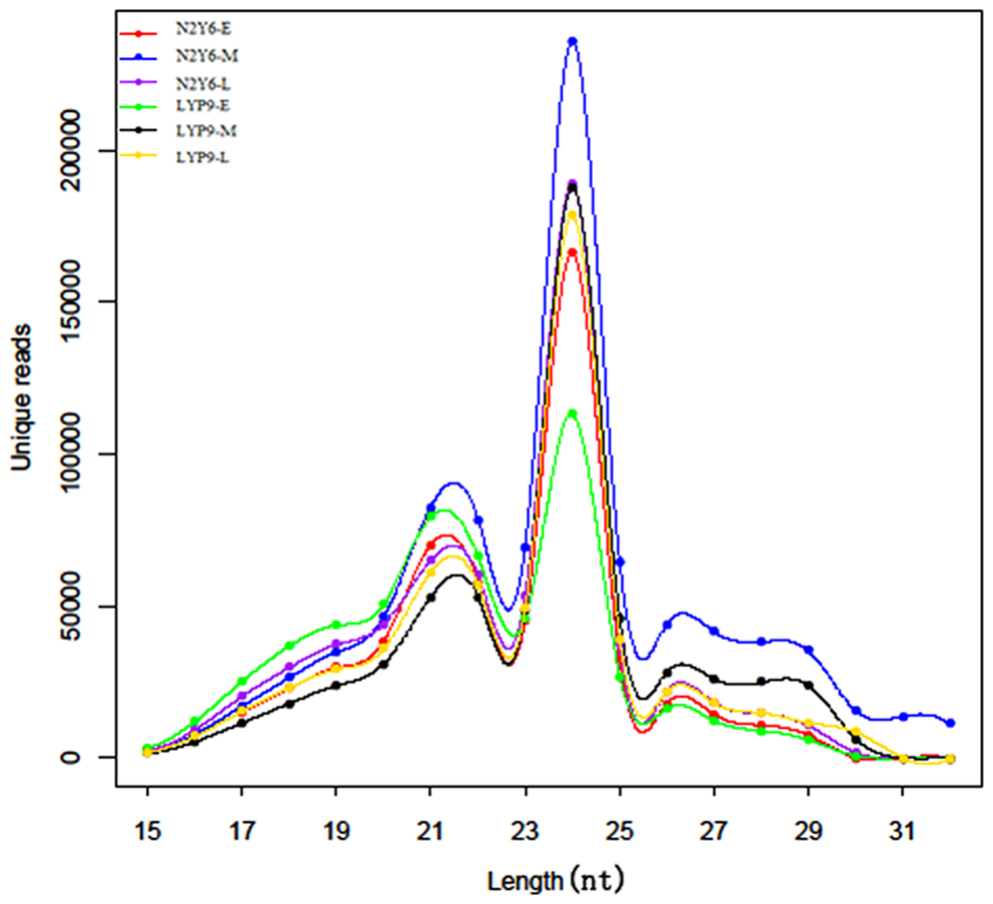
Length distribution of mappable counts of sequ-seqs type in six libraries of leaf.

To identify possible miRNA involved in the leaf senescence of N2Y6 and LYP9, six small RNA libraries from the flag leaves of N2Y6 and LYP9 were constructed and sequenced by high-throughput Illumina Solexa system, respectively. The number of raw reads yielded from the leaves in different stages of the grain-filling in N2Y6 and LYP9 were 7,285,774 and 7,727,107 at the early stage, 12,516,204 and 8,615,227 at the middle stage, and 8,412,408 and 5,726,539 at the late stage, respectively (Table 1). After removal of corrupted adapter sequences, reads with length <15 and > 25 nt, junk reads, common RNA families (mRNA, rRNA, tRNA, snRNA, snoRNA) and repeats, a total of 5,307,483 and 4,820,096 mappable reads for the early grain-filling stage, 8,369,566 and 5,326,457 ones for the middle stage, and 5,576,891 and 4,095,534 ones for the late stage were obtained, respectively (Table 1). The size distribution of the mappable reads did not show significantly difference among the six libraries. The majority of the reads were 20–24 nt, with 24 nt as the most frequent size followed by 21 nt as the second largest percentage representing the class of endogenous small RNA families (Fig. 2).

**Table 1:** Overview of reads from raw data to cleaned sequences.

	N2Y6-E		N2Y6-M		N2Y6-L		LYP9-E		LYP9-M		LYP9-L	
RNA class	counts	Percentage of total	counts	percentage of total	counts	percentage of total	counts	percentage of total	counts	percentage of total	counts	percentageof total
Raw reads	7285774	100	12516204	100	8412408	100	7727107	100	8615227	100	5726539	100
3ADT&length filter	458744	6.3	1569764	12.54	873723	10.39	979790	12.68	1751774	20.33	396886	6.93
Junk reads	11629	0.16	29910	0.24	12572	0.15	12890	0.17	20326	0.24	14498	0.25
Rfam	915420	12.56	1617769	12.93	1156692	13.75	1111146	14.38	976714	11.34	736977	12.87
mRNA	881815	12.1	1365424	10.91	1135910	13.5	1204102	15.58	833965	9.68	679048	11.86
Repeats	72900	1	93715	0.75	118700	1.41	109179	1.41	90416	1.05	52236	0.91
rRNA	477605	6.56	1059109	8.46	731464	8.7	697772	9.03	573906	6.66	393905	6.88
tRNA	340701	4.68	394924	3.16	281522	3.35	236105	3.06	301997	3.51	248862	4.35
snoRNA	17137	0.24	32249	0.26	26564	0.32	34106	0.44	20219	0.23	21762	0.38
snRNA	17280	0.24	31258	0.25	22750	0.27	29455	0.38	24359	0.28	23468	0.41
other Rfam RNA	62697	0.86	100229	0.8	94392	1.12	113708	1.47	56233	0.65	48980	0.86
Mappable reads	5307483	72.85	8369566	66.87	5576891	66.29	4820096	62.38	5326457	61.83	4095534	71.52

3ADT&length filter: reads removed due to 3ADT not found and length with <17 nt and>25 nt were removed.

Junk reads: Junk:> = 2N,> = 7A,> = 8C,> = 6G,> = 7T,> = 10Dimer,> = 6Trimer, or> = 5Tetramer.

Rfam: collection of many common non-coding RNA families except micro RNA; http://rfam.janelia.org.

Repeats: prototypic sequences representing repetitive DNA from different eukaryotic species; http://www.girinst.org/repbase.

Notes: there is overlap in mapping of reads with mRNA, rRNA, tRNA, snRNA, snoRNA and repeats.

### Identification and expression analysis of known miRNAs and their isoforms

To identify the known miRNAs in the six libraries, the clean reads were compared with the currently known plant precursor or mature miRNA sequences in miRBase 20.0 (http://www.mirbase.org/). A total of 592 pre-miRNAs corresponding to 713 mature miRNAs were found. Among them, 268 pre-miRNAs corresponding to 371 miRNAs, as well as 91 new mature 5′-or 3′-miRNAs corresponding to *O. sativa* pre-miRNAs were detected for the first time (Table S1). Moreover, many variants of the known miRNAs were identified, especially at the 3′ and 5′ ends. The variants were defined as “isomiRs”, which frequently had higher counts than the corresponding known reference miRNA sequences listed in miRBase. The most abundant sequence in alignment with known rice miRNAs listed in miRBase 20.0 was identified as a representative of the alignment and designated ‘‘dominant isomiR’’. A dominant isomiR was further defined based upon its differences from the reference miRNA sequence in miRBase 20.0. For example, osa-miR160a-5p_L+1R-1 (L = 5'end; R = 3'end) is a variant of osa-miR160a-5p and is 1 nt longer at 5' end and 1 nt shorter at 3' end than osa-miR160a-5p, and osa-miR812s_1ss11AG contains a variation from A to G at the 11th nucleotide compared with the reference osa-miR812s. In total, 28 known rice miRNA isomiRs had more counts than reference miRNA sequences listed in miRBase 20.0. The details of isomiRs for each known miRNA are presented in Table S1.

**Figure 3:**
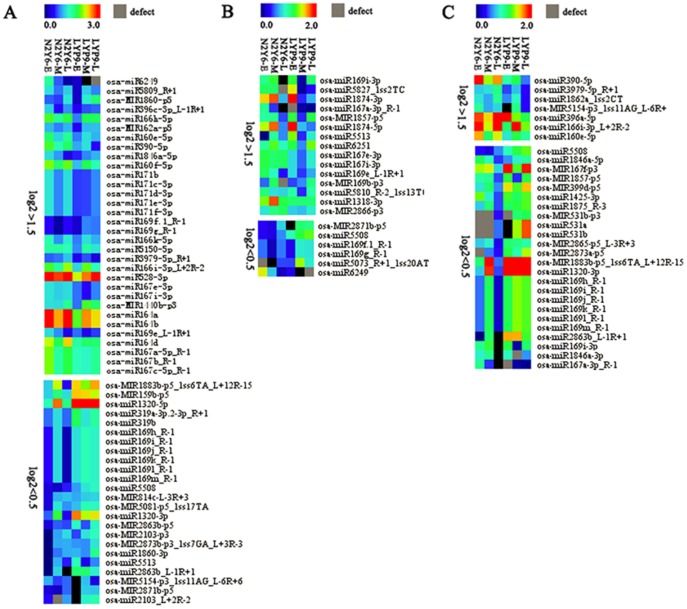
Differential expression levels of known miRNAs in the leaves of N2Y6 and LYP9. (A) miRNAs with the expression level of log_2_>1.5 and log_2_<0.5 in the leaves of N2Y6 at the early stage of grain-filling as compared with that in LYP9. (B) miRNAs with the expression level of log_2_>1.5 and log_2_<0.5 in the leaves of N2Y6 at the middle stage of grain-filling as compared with that in LYP9. (C) miRNAs with the expression level of log_2_>1.5 and log_2_<0.5 in the leaves of N2Y6 at the late stage of grain-filling as compared with that in LYP9.

To identify the known miRNAs that involved in senescence of leaf at early, middle and late stages of grain-filling in rice, differential expression of them in the six libraries was analyzed and compared using the counts of reads generated from the high-throughput sequencing (Table S2). Considering the extremely low abundances might lead to false results, the miRNAs with less than 10 norm reads were removed from the expression analysis. The expression of miRNAs with log_2_ fold changes higher than 1.5 was designated as up-regulated, and less than 0.5 was designated as down-regulated. Compared with the expression of miRNA in leaves of LYP9-E, LYP9-M and LYP9-L, the numbers of up-regulated and down-regulated known miRNAs were 32 and 24 for N2Y6-E, 15 and 6 for N2Y6-M, and 7 and 24 for N2Y6-L, respectively (Fig. 3, Table S2). Moreover, six miRNAs, including osa-miR160e-5p, osa-miR166i-3p_L+2R-2, osa-miR167e-3p, osa-miR167i-3p, osa-miR390-5p and osa-miR3979-5p_R+1, were up-regulated in leaves at least in two stages of grain-filling in rice N2Y6, and 11 miRNAs, including osa-miR1320-3p, osa-miR169h_R-1, osa-miR169i_R-1, osa-miR169j_R-1, osa-miR169k_R-1, osa-miR169l_R-1, osa-miR169m_R-1, osa-MIR1883b-p5_1ss6TA_L+12R-15, osa-miR2863b_L-1R+1, osa-MIR2871b-p5 and osa-miR5508, were down-regulated in leaves at least in two stages of grain-filling in rice N2Y6 (Table 2). Interestingly, compared with the expression in leaves of LYP9, osa-miR5508 was found to be down-regulated in all leaves of three stages of grain-filling in rice N2Y6 (Table 2), suggested that it might perform important role in regulating leaf senescence.

**Table 2:** The known and miRNA candidates which showed significant changes in leaves at least in two stages of grain-filling in the two hybrid rice.

miR_name	log_2_(N2Y6-E/LYP9-E)	log_2_(N2Y6-M/LYP9-M)	log_2_(N2Y6-L/LYP9-L)
osa-miR160e-5p	+ +	−	+ +
osa-miR166i-3p_L+2R-2	+ +	−	+ +
osa-miR167e-3p	+ +	+ +	−
osa-miR167i-3p	+ +	+ +	−
osa-miR390-5p	+ +	−	+ +
osa-miR3979-5p_R+1	+ +	−	+ +
PC-3p-337602_10	+ +	+ +	−
PC-3p-638008_4	+ +	+ +	−
PC-5p-151167_37	−	+ +	+ +
PC-5p-171396_32	+ +	−	+ +
osa-miR1320-3p	+	−	+
osa-miR169h_R-1	+	−	+
osa-miR169i_R-1	+	−	+
osa-miR169j_R-1	+	−	+
osa-miR169k_R-1	+	−	+
osa-miR169l_R-1	+	−	+
osa-miR169m_R-1	+	−	+
osa-MIR1883b-p5_1ss6TA_L+12R-15	+	−	+
osa-miR2863b_L-1R+1	+	−	+
osa-MIR2871b-p5	+	+	−
osa-miR5508	+	+	+
PC-5p-187365_25	+	−	+
PC-3p-269952_20	+	+	−
PC-3p-347190_9	+	−	+
PC-5p-413922_9	−	+	+
PC-5p-1293438_2	−	+	+

−: no significant changes.

+: log_2_<0.5.

+ +: log_2_>1.5.

### Identification and expression analysis of miRNA candidates

The ability of the miRNA flanking sequences to fold-back into a stable hairpin structure is an important criterion for the annotation of new miRNAs [20]. To identify miRNA candidates, all unique sRNA sequences were used to query the genome of indica rice 93-11 and PA64S (http://rise2.genomics.org.cn/page/rice/index.jsp), the male and female parents of LYP9. All genomic loci-generating sRNAs that can be folded into a secondary structure were considered as miRNA candidates. In total, 95 pre-miRNAs corresponding to 162 mature miRNAs originated from predicted RNA hairpins were first identified in rice, of which most were 21 and 24 nt in length (Table S3).

**Figure 4:**
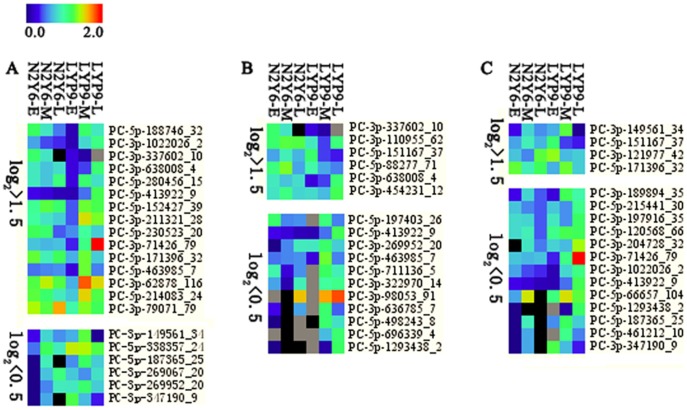
Differential expression levels of miRNA candidates in the leaves of N2Y6 and LYP9. (A) MiRNA candidates with the expression level of log_2_>1.5 and log_2_<0.5 in the leaves of N2Y6 at the early stage of grain-filling as compared with that in LYP9. (B) MiRNA candidates with the expression level of log_2_>1.5 and log_2_<0.5 in the leaves of N2Y6 at the middle stage of grain-filling as compared with that in LYP9. (C) MiRNA candidates with the expression level of log_2_>1.5 and log_2_<0.5 in the leaves of N2Y6 at the late stage of grain-filling as compared with that in LYP9.

To identify miRNA candidates that involved in leaf senescence, their expression in leaves at early, middle and late stages of grain-filling in two hybrid rice was analyzed and compared using the counts of reads generated from the high-throughput sequencing (Table S2). Similarly, the miRNA candidates with less than 10 norm reads were removed from the expression analysis. The expression of miRNAs with log_2_ fold changes higher than 1.5 was designated as up-regulated, and less than 0.5 was designated as down-regulated. Compared with the expression of miRNA in leaves of LYP9-E, LYP9-M and LYP9-L, the numbers of up-regulated and down-regulated miRNA candidates were 15 and 6 for N2Y6-E, 6 and 11 for N2Y6-M, and 4 and 13 for N2Y6-L, respectively (Fig. 4, Table S2). Moreover, four miRNA candidates, PC-3p-337602_10, PC-3p-638008_4, PC-5p-151167_37 and PC-5p-171396_32, were up-regulated in leaves at least in two stages of grain-filling in rice N2Y6, and five miRNAs, including PC-5p-187365_25, PC-3p-269952_20, PC-3p-347190_9, PC-5p-413922_9 and PC-5p-1293438_2, were down-regulated in leaves at least in two stages of grain-filling in rice N2Y6 (Table 2).

### MiRNAs expression profiling validation

**Figure 5:**
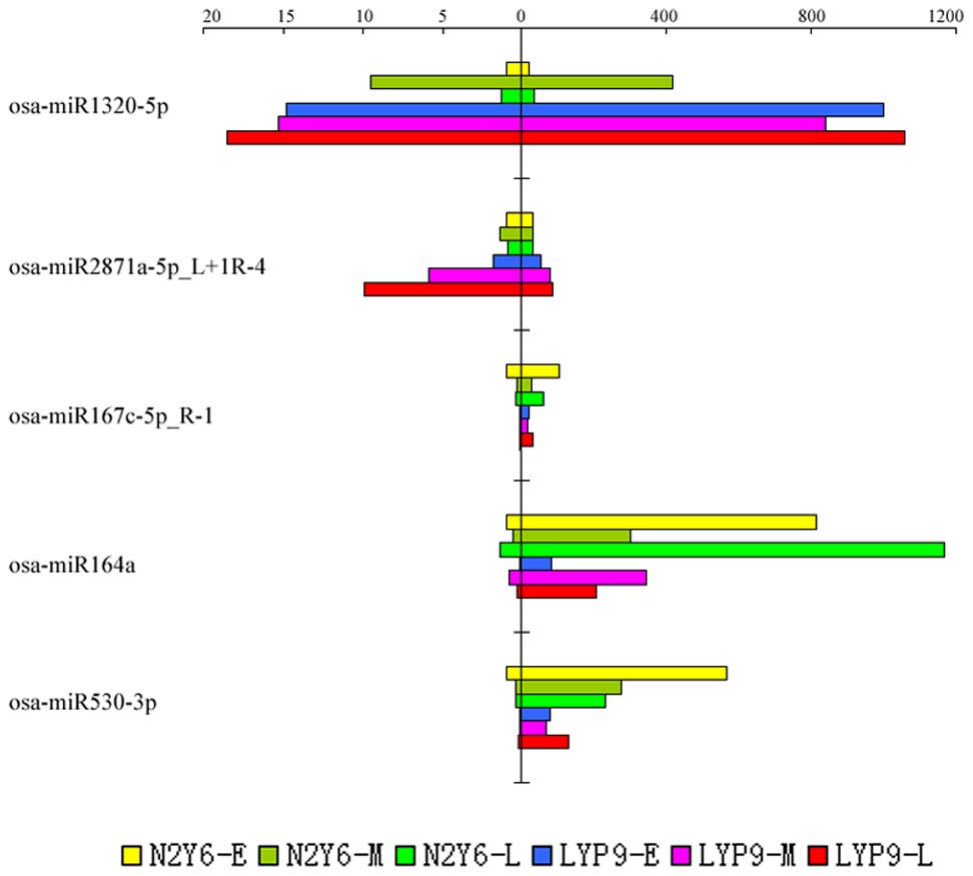
Expression validation of miRNAs in the leaves of N2Y6 and LYP9 by qRT-PCR. The amount of expression was normalized by the level of OsU6_snRNA in qRT-PCR. All reactions of qRT-PCR were repeated three times for each sample. The left indicates the miRNA relative expression tested by qRT-PCR, and the right indicates the miRNA relative expression generated from the high-throughput sequencing.

To validate the sequencing results and the expression level of miRNAs, the differential expression pattern of five miRNAs, osa-miR1320-5p, osa-miR164a, osa-miR167c-5p_R-1, osa-miR2871a-5p_L+1R-4 and osa-miR530-3p, which showed significant changes by sequencing in the two rice, were randomly selected for qRT-PCR analysis. As shown in Fig. 5, qRT-PCR results were consistent with the data from high-throughput sequencing for all the five miRNAs in all the three stages.

### Target genes of miRNAs by degradome sequencing

**Figure 6:**
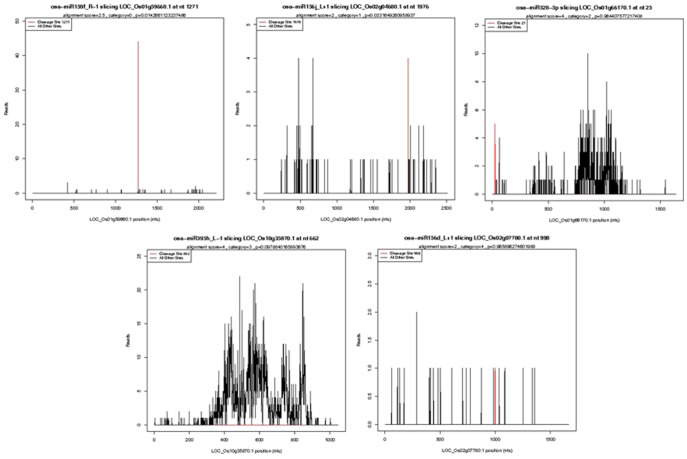
Five different categories of T-plot of miRNA targets. T-plots show the distribution of the degradome tags along the full-length of the target mRNA sequence. The red line represents the sliced target transcripts.

To date, limited targets for miRNAs have been identified in plants. To further understand the biological function of the miRNAs described above, we performed a genome-wide sequencing of miRNA-cleaved mRNA and Cleveland analysis based on high-throughput degradome sequencing technology. In total, 883 and 805 targets were identified in N2Y6 and LYP9, respectively (Table S5). Based on the signature abundance at the target sites, the cleaved targets were classified into categories 0, 1, 2, 3 and 4 (Fig. 6). Category ‘0’ is defined as > 1 raw read at the position, with abundance at a position equal to the maximum on the transcript, and with only one maximum on the transcript. Category ‘1’ is described as > 1 raw read at the position, with abundance at the position equal to the maximum on the transcript, and more than one maximum position on the transcript. Category ‘2’ includes > 1 raw read at the position and abundance at the position less than the maximum but higher than the median for the transcript. Category ‘3’ comprises the transcripts with > 1 raw read at the position, and abundance at the position equal to or less than the median for the transcript. Category ‘4’ showes only one raw read at the position. Of the 883 targets identified for N2Y6, 205 fell into category 0, 18 into category 1, 314 into category 2, 5 into category 3, and 341 into category 4, respectively (Table S5). Of the 805 targets identified for LYP9, 200 fell into category 0, 52 into category 1, 295 into category 2, 37 into category 3, and 221 into category 4 (Table S4).

Based on the BLASTX analysis, most of the targets detected in the two degradome libraries were identified (Table S5). Among them, many were reported to be involved in senescence, such as *auxin response factors* (*ARF*), *NAC transcription factor*, *APETALA2* (*AP2*)-*like factor*, *Myb transcription factors*, *WRKY transcription factor*, *Vacuolar-sorting receptor*, *subtilisin* and *scarecrow-like protein*, *Serine/threonine-protein kinase*, *Scl1 protein*, *dihydrofolate reductase*, *beta-glucosidase*, etc (Table 3).

**Table 3:** Parts of the identified targets involved in leaf senescence by degradome sequencing.

miRNA	Target	Alignment score	Alignment range	Cleavage site	Definition
osa-miR1429	LOC_Os08g42370.3	4	1156–1175	1166	putative DHHC-type zinc finger domain-containing protein
	LOC_Os08g42370.2	4	920–939	930	putative DHHC-type zinc finger domain-containing protein
osa-miR156	LOC_Os01g69830.1	1.5	1153–1173	1164	Squamosa promoter-binding-like protein 2
	LOC_Os02g04680.1	2	1965–1984	1975	Squamosa promoter-binding-like protein 3
	LOC_Os02g04680.2	2	1962–1981	1972	Squamosa promoter-binding-like protein 3
	LOC_Os02g07780.1	2	987–1007	998	Squamosa promoter-binding-like protein 4
	LOC_Os02g07780.2	2	2221–2241	2232	squamosa promoter binding protein-like
	LOC_Os06g49010.1	1	1686–1706	1697	Squamosa promoter-binding-like protein 12
	LOC_Os06g49010.2	1	1494–1514	1505	Squamosa promoter-binding-like protein 12
	LOC_Os06g49010.3	1	1439–1459	1450	Squamosa promoter-binding-like protein 12
	LOC_Os06g49010.4	1	1638–1658	1649	Squamosa promoter-binding-like protein 12
	LOC_Os06g49010.5	1	2018–2038	2029	Squamosa promoter-binding-like protein 12
	LOC_Os06g49010.6	1	1842–1862	1853	Squamosa promoter-binding-like protein 12
	LOC_Os07g32170.1	3	852–872	863	Squamosa promoter-binding-like protein 13
	LOC_Os07g32170.2	3	664–684	675	Squamosa promoter-binding-like protein 13
	LOC_Os08g41940.1	1	1054–1073	1064	Squamosa promoter-binding-like protein 16
osa-miR159	LOC_Os10g05230.2	4	335–355	346	Zinc finger
	LOC_Os01g59660.1	3.5	1260–1280	1271	myb proto-oncogene protein, plant
	LOC_Os01g59660.2	3.5	1348–1368	1359	myb proto-oncogene protein, plant
	LOC_Os01g59660.3	3.5	1258–1278	1269	myb proto-oncogene protein, plant
	LOC_Os01g59660.4	3.5	1180–1200	1191	myb proto-oncogene protein, plant
	LOC_Os06g40330.1	3.5	1418–1438	1429	myb proto-oncogene protein, plant
osa-miR160	LOC_Os02g41800.1	1	1484–1504	1495	Auxin response factor 8
	LOC_Os02g41800.2	1	1484–1504	1495	Auxin response factor 8
	LOC_Os04g43910.1	1	1344–1364	1355	Auxin response factor 10
	LOC_Os06g47150.1	0.5	2043–2063	2054	Auxin response factor 18
	LOC_Os06g47150.2	0.5	1845–1865	1856	Auxin response factor 18
	LOC_Os06g47150.3	0.5	1812–1832	1823	Auxin response factor 18
	LOC_Os06g47150.4	0.5	1845–1865	1856	Auxin response factor 18
	LOC_Os10g33940.1	0.5	1636–1656	1647	Auxin response factor 16
osa-miR164	LOC_Os08g10080.1	3.5	772–792	783	NAC domain-containing protein 21/22
	LOC_Os12g41680.1	2	911–931	922	salicylic acid-induced protein 19
osa-miR166	LOC_Os03g01890.1	3	1089–1109	1100	homeobox-leucine zipper protein
	LOC_Os03g01890.2	3	1089–1109	1100	homeobox-leucine zipper protein
	LOC_Os03g43930.1	3	955–975	966	homeobox-leucine zipper protein
	LOC_Os03g43930.2	3	955–975	966	homeobox-leucine zipper protein
	LOC_Os06g01304.2	4	1922–1942	1933	vacuolar protein 8
	LOC_Os06g01304.3	4	2017–2037	2028	vacuolar protein 8
	LOC_Os10g33960.1	3	924–944	935	homeobox-leucine zipper protein
	LOC_Os10g33960.2	3	696–716	707	homeobox-leucine zipper protein
	LOC_Os10g33960.3	3	924–944	935	homeobox-leucine zipper protein
	LOC_Os10g33960.4	3	924–944	935	homeobox-leucine zipper protein
	LOC_Os12g41860.1	3	877–897	888	homeobox-leucine zipper protein
osa-miR167	LOC_Os02g06910.1	4	3231–3251	3241	Auxin response factor 6
	LOC_Os06g46410.1	4	3195–3215	3205	Auxin response factor 17
	LOC_Os06g46410.2	4	2571–2591	2581	Auxin response factor 17
	LOC_Os09g38790.3	4	1150–1171	1162	Zinc finger protein 207
osa-miR171b	LOC_Os02g44360.1	1	1351–1371	1362	Scarecrow-like protein 1
	LOC_Os04g46860.1	1	1326–1346	1337	Scarecrow-like protein 6
	LOC_Os06g01620.1	1	456–476	467	Scarecrow-like protein 1
	LOC_Os06g01620.1	1	456–476	467	Scarecrow-like protein 6
osa-miR172a	LOC_Os03g60430.1	2	1755–1775	1766	AP2-like factor, euAP2 lineage
	LOC_Os03g60430.2	2	1767–1787	1778	AP2-like factor, euAP2 lineage
	LOC_Os04g55560.2	4	1623–1643	1634	AP2-like factor, euAP2 lineage
	LOC_Os04g55560.3	4	1634–1654	1645	AP2-like factor, euAP2 lineage
	LOC_Os05g03040.1	2	1976–1996	1987	AP2-like factor, euAP2 lineage
	LOC_Os05g03040.2	2	1552–1572	1563	AP2-like factor, euAP2 lineage
	LOC_Os05g03040.3	2	2089–2109	2100	AP2-like factor, euAP2 lineage
	LOC_Os07g13170.1	2	1404–1424	1415	AP2-like factor, euAP2 lineage
	LOC_Os07g13170.2	2	1463–1483	1474	AP2-like factor, euAP2 lineage
	LOC_Os09g21770.1	3	686–705	697	putative zinc-finger motif
osa-miR1848	LOC_Os10g39936.1	4	630–651	642	Zinc finger
	LOC_Os03g19020.2	4	564–583	574	PHD-finger family protein
	LOC_Os03g19020.1	4	658–677	668	PHD-finger family protein
	LOC_Os03g19020.3	4	564–583	574	PHD-finger family protein
osa-MIR1851	LOC_Os07g48260.1	3.5	1179–1198	1189	WRKY transcription factor 54
	LOC_Os03g49620.3	4	1803–1824	1815	Probable LRR receptor-like serine/threonine-protein kinase
osa-miR319	LOC_Os01g59660.1	4	1259–1279	1270	myb proto-oncogene protein, plant
	LOC_Os01g59660.2	4	1347–1367	1358	myb proto-oncogene protein, plant
	LOC_Os01g59660.3	4	1257–1277	1268	myb proto-oncogene protein, plant
	LOC_Os01g59660.4	4	1179–1199	1190	myb proto-oncogene protein, plant
osa-miR535	LOC_Os06g45310.1	4	852–873	863	Squamosa promoter-binding-like protein 11
osa-miR5809	LOC_Os04g47160.1	4	1685–1705	1696	subtilisin
	LOC_Os05g03760.1	4	1705–1725	1716	putative finger transcription factor
PC-3p-18725_264	LOC_Os01g09200.1	4	262–277	269	serine/threonine kinase 38
PC-3p-18730_326	LOC_Os09g29170.4	4	122–141	133	Serine/threonine-protein kinase PEPKR2
	LOC_Os09g29170.3	4	122–141	133	Serine/threonine-protein kinase PEPKR2
	LOC_Os09g29170.2	4	37–56	48	Serine/threonine-protein kinase PEPKR2
	LOC_Os09g29170.1	4	37–56	48	Serine/threonine-protein kinase PEPKR2
PC-3p-269067_20	LOC_Os11g44810.2	4	323–340	330	auxin-repressed protein-like protein ARP1
	LOC_Os11g44810.1	4	328–345	335	auxin-repressed protein-like protein ARP1
PC-3p-35500_181	LOC_Os06g45380.2	4	212–226	218	Vacuolar-sorting receptor 6
	LOC_Os06g45380.1	4	935–949	941	Vacuolar-sorting receptor 6
	LOC_Os12g06490.2	3.5	439–454	445	serine/threonine-protein kinase WNK1
	LOC_Os12g06490.1	3.5	477–492	483	serine/threonine-protein kinase WNK1
	LOC_Os01g25370.3	4	202–217	208	sentrin-specific protease 1
	LOC_Os01g25370.2	4	202–217	208	sentrin-specific protease 1
	LOC_Os01g25370.1	4	202–217	208	sentrin-specific protease 1
PC-3p-779969_7	LOC_Os06g41010.4	4	433–452	443	AN1-type zinc finger and ubiquitin domain-containing protein 1
	LOC_Os06g41010.3	4	251–270	261	AN1-type zinc finger and ubiquitin domain-containing protein 1
	LOC_Os06g41010.1	4	350–369	360	AN1-type zinc finger and ubiquitin domain-containing protein 1
PC-5p-151167_37	LOC_Os01g70270.4	3.5	2747–2765	2756	Auxin response factor 4
	LOC_Os01g70270.3	3.5	3109–3127	3118	Auxin response factor 4
	LOC_Os01g70270.2	3.5	2867–2885	2876	Auxin response factor 4
	LOC_Os01g70270.1	3.5	2843–2861	2852	Auxin response factor 4
PC-5p-215441_30	LOC_Os03g54780.1	4	1661–1676	1667	serine/threonine kinase 25
PC-5p-2331403_1	LOC_Os06g04970.1	3	1448–1471	1462	serine protease-like
PC-5p-284772_19	LOC_Os01g37960.1	4	1274–1289	1280	putative auxin amidohydrolase
PC-5p-705811_3	LOC_Os05g33940.1	4	1142–1157	1148	cell death associated protein
	LOC_Os07g48550.1	4	1243–1258	1249	NAC domain-containing protein 100

(More targets were shown in Table S6).

### GO function analysis of targets

**Figure 7:**
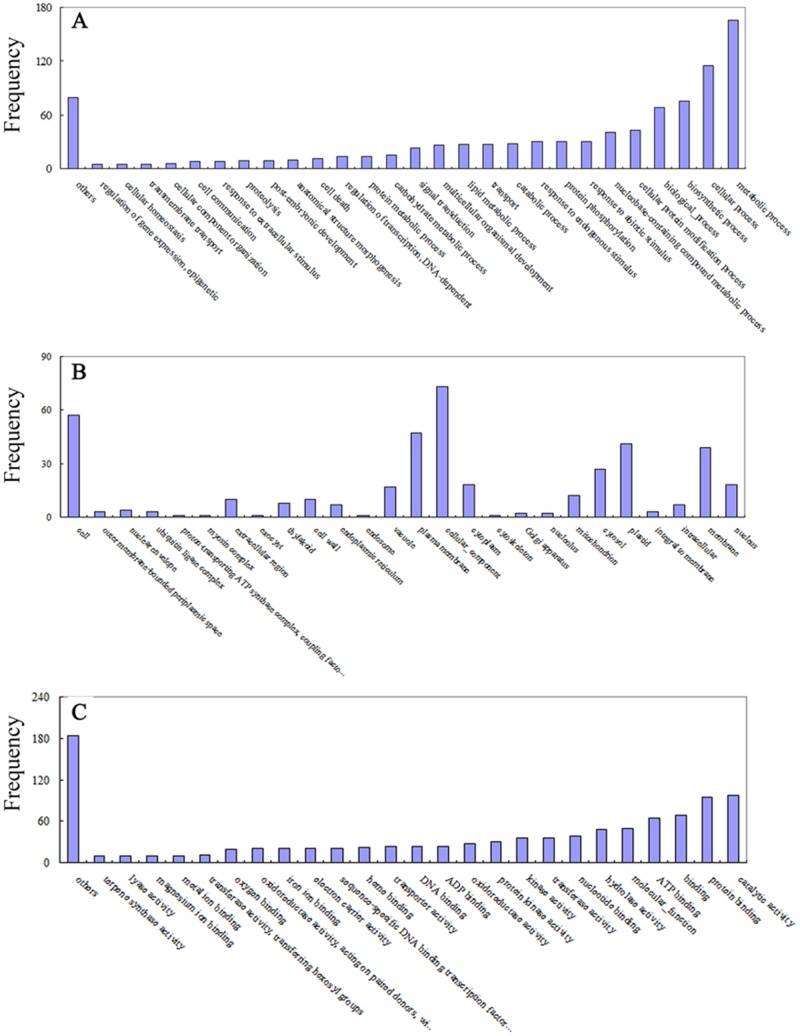
Distribution of the identified targets classified based on putative biological processes (A) cellular component (B), and molecular functions (C). The GO analysis was carried out according to *Arabidopsis thaliana* and *Oryza sativa* databases.

To help understand the miRNA-gene regulatory network, the identified target genes were subjected to Gene Ontology (GO) analysis based on the *A. thaliana* and *O. sativa* databases. Results showed that 1171 targets were involved in 81 biological processes (Fig. 7A), 413 targets involved in 26 kinds of cellular component (Fig. 7B), and 1018 targets involved in 99 kinds of molecular function (Fig. 7C). Among these targets, many have involved in plant senescence through phytohormone signaling pathways, For example, *AP2-like factor* and *zinc finger protein* involved in abscisic acid (ABA) signaling transduction, *ARF* involved in auxins and jasmonic acid (JA) biosynthesis and signaling transduction.

### Differentially expression of mRNA

**Figure 8:**
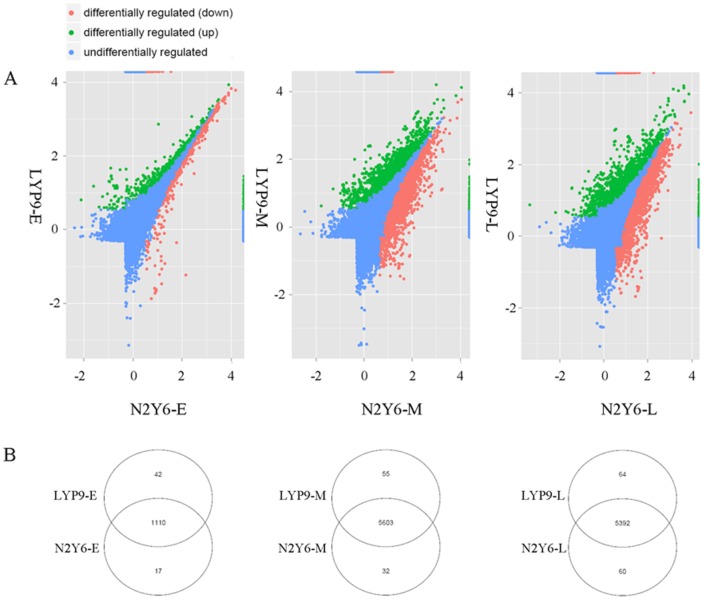
mRNAs expression in the leaves of N2Y6 and LYP9. (A) mRNAs expression level in the leaves of N2Y6 at the early, middle and late stages of grain-filling as compared with that in LYP9. (B) mRNA expression pattern in the leaves of N2Y6 and LYP9 at the early, middle and late stages of grain-filling.

**Figure 9:**
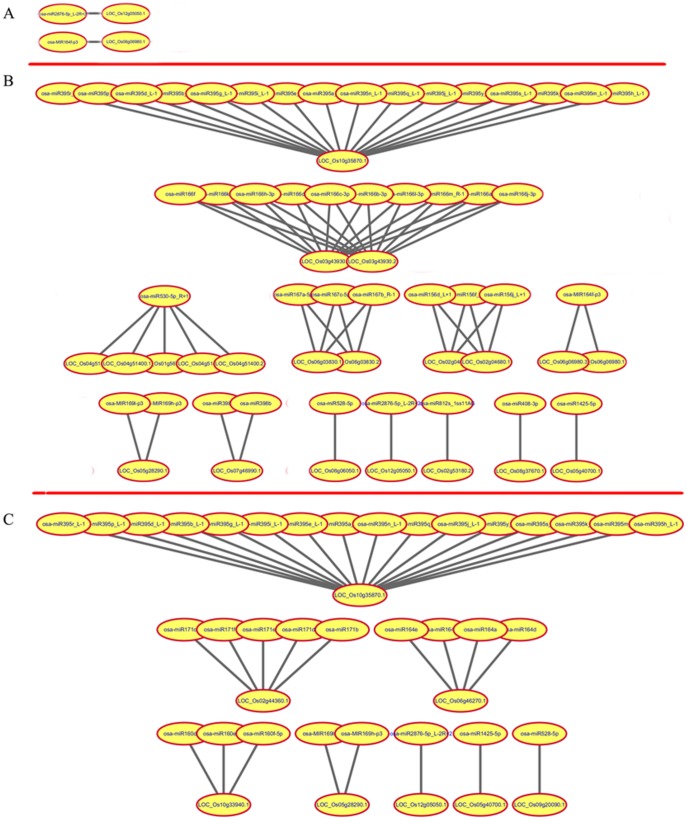
Targets of miRNAs showing significant expression changes in the leaves of N2Y6 and LYP9. (A) Targets in the leaves at the early stage of grain-filling; (B) Targets in the leaves at the middle stage of grain-filling; (C) Targets in the leaves at the late stage of grain-filling.

To investigate the gene expression changes in the leaves at early, middle and late stages of grain-filling of N2Y6 and LYP9, six RNA-seq tag libraries were constructed and sequenced by Illumina HiSeq 2000. Approximately 5–10 million raw tags were generated for each RNA-seq library, and more than 90% in each library were clean tags. The statistics of DGE tags as showed in Table S6, a total of 38,863 tags were identified. Compared with the expression of genes in leaves of LYP9-E, 1189 were up-regulated (787 genes) or down-regulated (402 genes) in leaves of N2Y6-E (Fig. 8A, Table S7). Among these 1189 genes, 42 expressed only in LYP9, 17 expressed only in N2Y6, and 1110 expressed in both LYP9 and N2Y6 (Fig. 8B, Table S7). Two genes, LOC_Os06g06980.1 and LOC_Os12g05050.1, identified to be targeting by osa-MIR164f-p3 and osa-miR2876-5p_L-2R+2 (Fig. 9A), respectively, showed significant changes between leaves of LYP9-E and N2Y6-E (Table S7). Compared with the expression of genes in leaves of LYP9-M, 5690 were up-regulated (2118 genes) or down-regulated (3572 genes) in leaves of N2Y6-M. Among these 5690 genes, 55 expressed only in LYP9, 32 expressed only in N2Y6, 5603 expressed in both LYP9 and N2Y6, respectively (Fig. 8A, B, Table S7), and 21 target genes showed significant changes between leaves of LYP9-E and N2Y6-E (Fig. 9B). Compared with the expression of genes in leaves of LYP9-L, 5516 were up-regulated (2135 genes) or down-regulated (3381 genes) in leaves of N2Y6-L, among which 64 expressed only in LYP9, 60 expressed only in N2Y6, and 5392 expressed in both leaves of LYP9-L and N2Y6-L (Fig. 8A, B, Table S7). Among these 5516 genes, only 33 target genes of miRNAs were found and 8 targets showed significant changes between leaves of LYP9-E and N2Y6-E (Fig. 9C, Table S7).

It was shown that the expression level of most of the identified targets in the six RNA-seq tag libraries were opposite with the miRNAs (Table S6). For example, in leaves at early, middle and late stages of grain-filling, the counts of reads of osa-miR159b generated from the high-throughput sequencing were 5,780, 9,100 and 6,103 in N2Y6, and were 25,694, 21,968, 27,743 in LYP9, respectively. Its target LOC_Os01g59660.1 were 3.2, 9.5, 8.2 in N2Y6, and were 3, 13, 17 in LYP9, respectively, target LOC_Os01g59660.2l, LOC_Os01g59660.3 and LOC_Os01g59660.4 were 4, 8, 3 in N2Y6, and 14, 18, 12 in LYP9, respectively, target LOC_Os06g40330.1 were 33,78, 26 in N2Y6, and were 31, 51, 38 in LYP9, respectively (Table S6). These data provided important convenience for us to analyze the senescence related miRNAs and targets, and demonstrated the important negative role of miRNAs in regulating the targets transcription in plant.

## Discussion

Functioning in transcriptional and post-transcriptional regulation of gene expression, miRNAs are involved in diverse developmental and physiological processes in plants [5]-[7]. Nevertheless, only few have been reported for their functions in leaf senescence [10]-[12]. To help understand the complex mechanism of leaf senescence in rice, miRNAs and their targets involved in leaf senescence were identified and analyzed in the present study.

ABA is an important phytohormone. In addition to the key roles on plant growth and responses to environmental stresses [21]-[24], it plays an important role in promoting leaf abscission and senescence [25]. The level of ABA increases in senescing leaves. Exogenous application of ABA could promote leaf senescence and induce expression of SAGs [26], [27]. The correlation between ABA and senescence was confirmed by the up-regulation of both ABA and leaf senescence under environmental stresses [28]. In plant responses to abiotic stresses, both ABA-dependent and ABA-independent pathways have been reported [29], [30]. Many transcription factors and their cognate cis-regulatory elements that function in either pathway have been identified [30], [31]. AP2, a group of transcription factors involved in plant responses to abiotic stresses through the ABA-dependent pathway, have been demonstrated to play important roles in plants senescence. In oil palm, the expression of EgAP2.1 in mesocarp was induced in response to ethylene and ABA, which was involved in fruit ripening and senescence [32]. In tomato, the *AP2a* gene negatively regulated fruit ripening and senescence [33]. MiR172b is a critical regulator specifically controlling *Arabidopsis* cotyledon greening during post-germinative growth by directly targeting SNZ under ABA treatment and osmotic stress [34]. In the present study, three members of the miR172 family targeting genes of AP2-like factors, osa-miR172a, osa-miR172c and osa-miR172d, were identified in the leaves of rice (Table 3, Table S5). In the leaves at early and middle stages of grain-filling, the expression of osa-miR172a, osa-miR172c and osa-miR172d was significantly higher in the resistant cultivar N2Y6 than in the senescence-susceptible cultivar LYP9 (Table S2), decreasing the expression of *AP2-like factor* gene (Table S6), by which leaf senescence of N2Y6 could be delayed.

Zinc finger protein also involves many aspects of plant growth and development, and plays important roles in responses to abiotic stresses through the ABA-dependent pathway. In *Arabidopsis*, tandem zinc finger 1 (*AtTZF1*) was reported to shuttle between the nucleus and cytoplasmic foci and function in ABA/GA-mediated growth and abiotic stress responses [35], [36]. *OsTZF1*, a homolog of *AtTZF1* in rice [35], acts as a negative regulator of leaf senescence under stress conditions and confers abiotic stress tolerance by delaying stress-response phenotypes. Another zinc finger protein, OsDOS, was also reported to be involved in delaying leaf senescence [37]. In the present study, several members of zinc finger transcription factor, targeted by osa-miR1848, osa-miR159b, osa-miR159a.1 and osa-MIR167f-p3, were identified (Table S1, Table S5). MiR159 has been indicated to determine leaf structure by targeting *MYB* in ToLCNDV infected tomato plants [38]. MiR167 has been shown to cleave *ARF8* in cultured rice cells,and involved in auxin signaling pathway [39]. In the leaves at early, middle and late stages of grain-filling, the expression of osa-miR159b, osa-miR159a.1 and osa-MIR167f-p3 was significantly lower in N2Y6 than in LYP9 (Table S2), increasing the expression of zinc finger gene and zinc finger protein 207 (Table S6), by which leaf senescence of N2Y6 could be delayed. This may be one of the mechanisms of osa-miR159, osa-miR167 and osa-miR172 mediated senescence-resistance through ABA-dependent pathway.

SA also play important role in leaf senescence. In the leaves undergoing senescence, the endogenous level of SA is increased by four times [40]. *Arabidopsis* mutants *npr1* and *pad4*, defective in SA signalling, exhibit reduced expression of a number of *SAGs* such as *PR1a*, *chitinase*, and *SAG12* [41], [42]. Leaves from *pad4* mutants do not appear to undergo cell death as efficiently as the wild type [43] and often remain yellow during the senescence stage [41], [44], showing a clear involvement of the SA pathway in leaf senescence. In the present study, four members of the miR164 family, osa-miR164a, osa-miR164b, osa-miR164d, and osa-miR164e were identified (Table S1), targeting one of SA pathway relative gene, *salicylic acid-induced protein 19* (*SIP19*) (Table S5). Compared with that in the leaves of LYP9, In leaves at early, middle and late stages of grain-filling, the expression of sa-miR164a, osa-miR164b osa-miR164d and osa-miR164e was significantly higher in N2Y6 than in LYP9 (Table S2), decreasing the expression of *SIP19* (Table S6), by which leaf senescence of N2Y6 could be delayed. This may be one of the mechanisms of osa-miR164 mediated senescence-resistance in rice.

As one of the largest transcription factor families in plants, the NAC transcription factors were involved in diverse processes and plant senescence. In *Arabidopsis* leaf, about 20 NAC transcription factors differentially regulated senescence [45]. Overexpression of the NAP gene, one of *Arabidopsis* NAC transcription factors, induced the premature senescence. Deficient mutation of the NAP gene delayed the leaf senescence of *Arabidopsis*, which could be restored by the NAP homologs from rice and kidney bean [46]. ANAC092/NAC2/ORE1 plays an important role in regulating leaf longevity [10] and salt-promoted senescing process [47], [48]. Moreover, EIN2 is a positive regulator of senescence [10], loss-of-function of EIN2 could delay leaf senescence process [49]. Recent study in *Arabidopsis* also showed that EIN3 works downstream of EIN2, represses miR164 transcription by binding to its promoters, induces *NAC2* expression and then advances leaf senescence [50]. In the present study, four members of miR164 family, osa-miR164a, osa-miR164b, osa-miR164d, and osa-miR164e, were found to target *NAC1* and *NAC21/22* (Table S5). The higher expression of osa-miR164a, osa-miR164b, osa-miR164d and osa-miR164e in the leaves at early, middle and late stages of grain-filling of N2Y6 decreased the expression of *NAC1* and *NAC21/22* (Table S2, Table S6), by which leaf senescence of N2Y6 could be delay. These may be another mechanism of osa-miR164 mediated senescence-resistance in rice.

As a plant development hormone, auxins play a negative role in leaf senescence [51]. Exogenous application of auxins could down-regulate the expression of SAGs and delay leaf senescence [51]–[53]. ARF are transcription factors that bind to TGTCTC auxin response elements (AuxRE) in promoters of auxin response genes and regulate their expression [54], [55]. In *Arabidopsis* leaves, *ARF2*, *ARF7* and *ARF*19 transcripts increased moderately, and *ARF1* transcripts decreased slightly in response to dark induced senescence [56]. The *arf2* single mutants can delay leaf senescence, and *arf1 arf2* double mutants enhanced this phenotype [56]. Negative regulation of *ARF10* by miR160 also plays a critical role in seed germination and leaf development, which at least in part by the mechanism of interactions between ARF10-dependent auxin and ABA pathways. Transgenic plants expressing miR160-resistant form of *ARF10*, which has silent mutations in the miRNA target site, results in over-accumulation of the transgene mRNA and the serrated and narrow leaves [57]. In the present study, seven members of *ARF*, including *ARF4*, *ARF6*, *ARF8*, *ARF10*, *ARF16*, *ARF17* and *ARF18*, targeted by os-miR160 (osa-miR160a-5p_L+1R-1, osa-miR160b-5p_L+1R-1, osa-miR160c-5p_L+1R-1, osa-miR160d-5p and osa-miR160e-5p and osa-miR160f-5p), osa-miR167 (osa-miR167a-5p_R-1, osa-miR167b_R-1 and osa-miR167c-5p_R-1) and PC-5p-151167_37, were identified (Table S5). In the leaves at early, middle and late stages of grain-filling, the expression of osa-miR160a-5p_L+1R-1, osa-miR160b-5p_L+1R-1, osa-miR160c-5p_L+1R-1, osa-miR160d-5p and osa-miR160e-5p was significantly lower in N2Y6 than in LYP9 (Table S2), which increased the expression of *ARF8*, *ARF10*, *ARF16* and *ARF18* (Table S6), and might have been involved in the leaf senescence.

JA, MeJA, and other derivatives, together known as jasmonates, play key roles in plant senescence. JA-dependent senescence is defective in the JA-insensitive mutant *coronatine insensitive 1* (*coi1*) [47]. Exogenous application of JA could increase expression of SAGs and stimulate leaf senescence [42]. The biosynthesis of JA was regulated by two members of the *ARF*, *ARF6* and *ARF8*. The *arf6* and *arf8* single mutation delayed flowering development, leading to the formation of seedless and parthenocarpic fruit [58]. In the present study, *ARF6* and *ARF8*, targeted by osa-miR167a-5p_R-1, osa-miR167b_R-1, osa-miR167c-5p_R-1, osa-miR160d-5p, osa-miR160e-5p and osa-miR160f-5p, were identified (Table S5). In the leaves at early, middle and late stages of grain-filling, the expression of osa-miR167a-5p_R-1, osa-miR167b_R-1, osa-miR167c-5p_R-1, osa-miR160d-5p, osa-miR160e-5p and osa-miR160f-5p was significantly higher in N2Y6 than in LYP9 (Table S2), decreasing the expression of *ARF6* and *ARF8* (Table S6), by which the production of JA could be repressed. This may be one of the mechanisms of osa-miR167 mediated senescence-resistance through JA pathway.

In addition, many other targets, such as WRKY transcription factor 54, Vacuolar-sorting receptor 6, subtilisin, myb and scarecrow-like protein, targeted by osa-miR1851, PC-3p-35500_181, osa-miR5809, osa-miR319, osa-miR159 and osa-miR171, were reported to be involved in senescence [59]-[62] and were identified in the present study (Table 3, Table S5). Whether they are involved in leaf senescence in rice, is also worthy to determine in the further research.

In conclusion, we found six miRNA families, osa-miR159, osa-miR160, osa-miR164, osa-miR167, osa-miR172 and osa-miR1848, were involved in the leaf senescence through phytohormone signaling pathway in rice. Although the mechanism how these miRNA delay or hasten the process of leaf senescence is not well known, our results provided valuable information for understanding the miRNA-mediated leaf senescence in rice and offered an important foundation for rice breeding. Further research is needed to identify the detailed mechanism of rice leaf senescence mediated by miRNA.

## Materials and Methods

### Plant materials

The flag leaves at early, middle and late stages of grain-filling of two hybrid rice, N2Y6 and LYP9 (named as N2Y6-E, N2Y6-M, N2Y6-L, LYP9-E, LYP9-M and LYP9-L, respectively), were collected, respectively. In each case, samples were pooled from twelve individual plants, immediately frozen in liquid nitrogen and stored at −80°C.

### Chlorophyll content detection

The content of chlorophyll a and chlorophyll b in leaves of the two hybrid rice was measured according to the methods of Shen et al. [63].

### Total RNA isolation, small RNA library preparation and sequencing

Total RNAs were extracted using the Trizol reagent (Life Technology, USA) according to the manufacturer's protocol. Total RNA quantity and purity were assayed with the NanoDrop ND-1000 spectrophotometer (Nano Drop) at 260/280 nm (ratio > 2.0). The total RNAs were 5′ and 3′ RNA adapter-ligated by T4 RNA ligase. The adapter-ligated small RNAs were transcribed to cDNA by Super-Script II Reverse Transcriptase and amplified using primers that annealed to the ends of the adapters. The cDNA libraries were obtained by 16% TBE gel and then subjected to Solexa/Illumina sequencing (LC Sciences).

### Analysis of sequencing data

Raw sequencing reads were processed into clean full-length reads by the LC Sciences small RNA pipeline (ACGT V4.2). Unique small RNAs were then used to query the mRNA (ftp.jgi-psf.org/pub/compgen/phytozome/v9.0/Osativa/annotation/), noncoding RNA sequences database (ftp://ftp.sanger.ac.uk/pub/databases/Rfam/10.1/) and the repeat-Repbase (http://www.girinst.org/repbase/update/index.html). New candidate miRNAs were identified by folding the flanking genome sequence of unique small RNAs, followed by the prediction of secondary structures by Mfold program. Differentially expressed miRNAs in the leaves of N2Y6 and LYP9 were identified by their counts of reads. The selection methods of differential expression were t-test, with the threshold of 0.05. Finally, all data were submitted to the database (http://www.ncbi.nlm.nih.gov/geo).

### Degradome sequencing and analysis

To investigate the potential target mRNAs involved in leaf senescence, two degradome cDNA libraries were constructed using sliced ends of polyadenylated transcripts from the leaves (mixture of leaves at early, middle and late stages of grain-filling of two hybrid rice, respectively) of N2Y6 and LYP9. Total RNA was extracted using Trizol reagent (Invitrogen, CA, USA) following the manufacturer's procedure. The total RNA quantity and purity were analysis of Bioanalyzer 2100 and RNA 6000 Nano LabChip Kit (Agilent, CA, USA) with RIN number >7.0. Approximately 20 ug of total RNA were used to prepare degradome library. The method of Ma et al [64] was followed with some modification. (1) Approximately 150 ng of poly(A)+ RNA was used as input RNA and annealing with biotinylated random primers. (2) Strapavidin capture of RNA fragments through biotinylated random primers. (3) 5′adaptor ligation to only those RNAs containing 5′-monophosphates. (4) Reverse transcription and PCR (5) Libraries were sequenced using the 5′adapter only, resulting in the sequencing of the first 36 nucleotides of the inserts that represented the 5′ends of the original RNAs. And then we performed the single-end sequencing (36 bp) on an Illumina Hiseq2500 at the LC-BIO (Hangzhou, China) following the vendor's recommended protocol. The purified cDNA library was used for cluster generation on Illumina's Cluster Station and then sequenced on Illumina Hiseq 2500 following vendor's instruction for running the instrument. Raw sequencing reads were obtained using Illumina's Pipeline v1.5 software following sequencing image analysis by Pipeline Firecrest Module and base-calling by Pipeline Bustard Module. The extracted sequencing reads were used in the standard data analysis. A Public software package, CleaveLand3.0 was used for analyzing sequencing data generated.

### Library construction, sequencing and data analysis for digital gene expression (DGE)

Six individual RNA-seq libraries of samples were constructed. Sequence library were prepared following the manufacturer's instructions. After 15 cycles of linear PCR amplification, around 250 bp fragments were purified by 6% TBE polyacrylamide gel electrophoresis to obtain the final tag libraries. Sequencing was performed using an Illumina HiSeq 2000. Millions of raw reads with a sequencing length of 35 bp were generated. The adaptors, empty tags (no tag sequence between the adaptors), low quality tags (tags containing one or more unknown nucleotides ‘‘N’’) and tags with a copy number of 1 were removed from the raw data to obtain the clean tags. All clean tags were mapped to the transcriptome reference database generated by RNA-Seq. The number of unambiguous tags corresponding to each gene was calculated and normalized to the RPKM (number of transcripts per million clean tags per kilobase) to analyze the expression of different genes. To identify genes expressed differentially between the two strains samples, an algorithm was developed based on the method described by Audic and Claverie. FDR (false discovery rate) was utilized to determine the threshold of P-values. "FDR #0.001 and the absolute value of the log2Ratio $1" were selected as the threshold for judging the diffrentially-expressed genes. A P-value of 0.05 was selected as the threshold for deciding whether a gene set was significantly enriched in GO and KEGG enrichment analysis.

### Real-time quantitative-PCR

The expression of five selected miRNAs were assayed in two lines of rice by Platinum SYBR Green-based q-PCR (Invitrogen, 11733–038) with the High Specificity miRNA QuantiMir RT Kit (RA610A-1, System Biosciences) on ABI 7900. Primers for the five miRNAs and internal control gene U6 snRNA are listed in Table S8.

### Accession number

Sequencing data obtained in this work have been submitted to the Gene Expression Omnibus under the accession number GSE62200.

## Supporting Information

Table S1 **Profile of the known miRNAs in rice leaves referred to miRbase20.0.**
(XLS)

Table S2 **Expression profiling of all the microRNAs discovered in rice leaves.**
(XLS)

Table S3 **Profile of potential miRNA candidates originating from predicted RNA hairpins**
(XLS)

Table S4 **Category of miRNAs targets in the leaves of N2Y6 and LYP9 at different stages of grain-filling.**
(XLS)

Table S5 **Profile of miRNA targets identified in the leaves of N2Y6 and LYP9.**
(XLS)

Table S6 **Differential expression of mRNAs in the leaves of N2Y6 and LYP9 at different stages of grain-filling.**
(XLS)

Table S7 **Differential expression of targets in the leaves of N2Y6 and LYP9 at different stages of grain-filling.**
(XLS)

Table S8 **Primers of miRNA used in this study.**
(XLS)
